# “The antibiotic paradox” in allogeneic stem cell transplantation

**DOI:** 10.1038/s41409-025-02706-y

**Published:** 2025-09-04

**Authors:** Daniela Weber, Elisabeth Meedt, Sakhila Ghimire, Andreas Hiergeist, Michael A. G. Kern, Matthias Höpting, Erik Thiele Orberg, Haroon Shaikh, Andreas Beilhack, Daniel Wolff, Matthias Edinger, Wolfgang Herr, Andre Gessner, Hendrik Poeck, Ernst Holler

**Affiliations:** 1 Clinic and Polyclinic for Internal Medicine III, University Hospital Regensburg, Regensburg, Germany; 2 Institute of Micrbiology and Hygiene, University Hospital Regensburg, Regensburg, Germany; 3 Experimental Stem Cell Transplantation. Departments of Internal Medicine II and Department of Pediatrics, University Hospital Würzburg, Würzburg, Germany; 4 Leibniz Institute for Immunotherapy (LIT), Regensburg, Germany

**Keywords:** Translational research, Bone marrow transplantation

## To the Editor:

In allogeneic stem cell transplantation (ASCT), antibiotic treatment of neutropenic and organ infections is widely used and needed: Blood stream infections (BSI) account for the most severe manifestations and occur either during neutropenia or are associated with organ complications such as acute gastrointestinal (GI) Graft-versus-Host Disease (GvHD) or pneumonia. Patients’ neutropenia and immunodeficiency demand immediate diagnosis and rapid empiric treatment until microbial culture results allow adapted treatment [1]. BSI affect between 13 and 30% of transplant recipients, markedly increasing mortality [2]. Due to the high BSI risk and the uncertain etiology of fever in the early transplant period, broad spectrum antibiotic treatment is started usually at the time of febrile neutropenia or fever of unknown origin (FUO) [3]. Current guidelines recommend de-escalation and discontinuation of antibiotics during neutropenia once infections clear [4]. Yet, a recent European survey, found only 36% of centers stop antibiotics before neutrophil recovery, resulting in long-term application.

With the introduction of 16 s rRNA sequencing refined microbiota analyses showed, that this early and prolonged use of antibiotics resulted in intestinal dysbiosis with loss of commensal, protective bacteria, abundance of facultative pathogens such as *Enterococci* [5] as well as resistant pathogens such as *Klebsiella, E. coli* and *Pseudomonas* [6]. Dysbiosis induces increased translocation and inflammation and patients who developed dysbiosis consistently experience worse outcomes compared to patients with diverse microbiota at neutrophil engraftment indicating the double-edged nature of early antibiotic treatment. The poorer outcome is mainly explained by an elevated risk of severe acute GvHD. Mechanistically, losing metabolites such as short chain fatty acids (SCFA) and indoles increases epithelial damage [7, 8] and undermines immunoregulation e.g., by regulatory T cells and thus explain this observation. In this context, changes of 3-indoxyl sulfate, indole acetate, indole acetylglutamine, and indole propionate, may limit tolerogenic indoleamine 2,3-dioxygenase (IDO) induction and thereby affect allogeneic T cell reactivity [9].

**Fig. 1:**
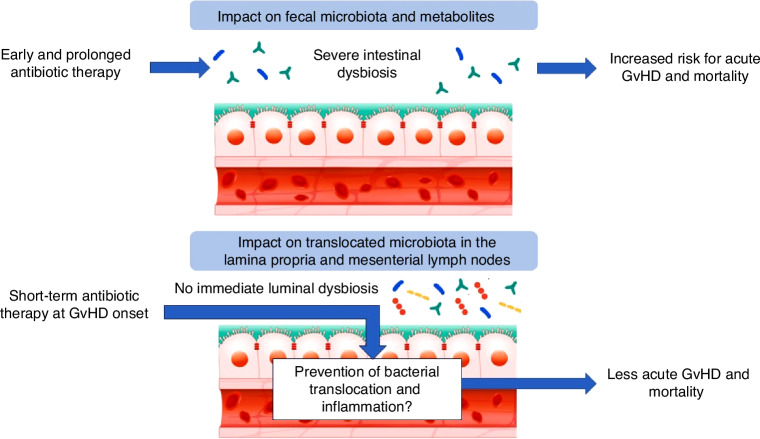
The antibiotic paradox explained by differential microbial targets: Early and prolonged antibiotic therapy affects luminal microbiota composition resulting in severe intestinal dysbiosis and an increased risk for acute GI GvHD and associated mortality (evidence by Weber et al. [15] and Peled et al. [5]). Antibiotic administration at GvHD onset suppresses bacterial translocation as important co-stimulator of T cell activation and may dampen GvHD (evidence by van Bekkum et al. [10], Schwab et al. [11]).

These findings although reproduced now in several analyses are confusing as they seem contradictory to the old observation of protection from acute GvHD by performing ASCT in germfree animal models [10] or by achieving complete decontamination especially in children, and we and other clinicians call it an *antibiotic paradox*. This paradox partly arises from the dual targets of antibiotic treatment. While antibiotics harm luminal microbiota and may induce mucus degradation, they also suppress and eliminate translocated bacteria that trigger GvHD. Intensive conditioning damages the epithelium, facilitating bacterial translocation – a hallmark of GvHD – which is amplified after loss of epithelial barrier integrity due to T cell mediated apoptosis seen in almost all patients suffering from GvHD; histologically, neutrophil infiltration is an indicator of bacterial translocation, leading to massive augmentation of T cell mediated damage by innate immune cell activation and inflammation [11]. In GvHD mice, living bacteria can be easily isolated from mesenteric lymph nodes, and patients with GI GvHD exhibit higher BSI rates, confirming the role of bacterial translocation. Thus, it is not surprising that murine models frequently report beneficial antibiotic effects because they cannot disentangle the impacts of dysbiosis and translocation due to overlapping kinetics (Fig. 1).

This *antibiotic paradox* explains why antibiotics can either enhance or suppress T-cell mediated immunity, depending on the context: While dysbiosis erodes T-cell regulation and increases GvHD susceptibility, suppression of translocated bacteria dampens T-cell activation through reduced co-stimulation mitigating GvHD. More broadly, antibiotics may blunt beneficial immune reactions induced by checkpoint inhibitors or CAR T-cells. An imbalanced gut microbiome contributes to primary resistance against immune checkpoint inhibitors, a condition worsened by antibiotics, whereas restoring microbial diversity through fecal microbiota transplantation or application of bacterial consortia improves clinical response [12].

A similar pattern was observed in patients treated with CAR-T cells, where clinical response was strongly correlated with a decrease in microbiome diversity and a subsequent systemic dysmetabolome, which was induced by prior use of broad-spectrum antibiotics with extensive anaerobic coverage, such as piperacillin-tazobactam and meropenem [13]. This is likely due to the role of SCFAs in modulating metabolic fitness and epigenetic imprinting thus impacting CAR T cell differentiation and efficacy. Of note, initiation of broad-spectrum antibiotics after CAR-T infusion with an exposure of less than 10 days did not appear to compromise anti-lymphoma treatment efficacy.

Translating these findings into clinical practice remains challenging. Adhering to antibiotic stewardship and following de-escalation guidelines may reduce the antibiotic exposure, thereby minimizing the risk of dysbiosis. Improved diagnostic strategies to distinguish FUO from fever due to cytokine release syndrome (CRS) could restrict early antibiotic use as CRS typically occurs after the application of (i) CAR T cells, (ii) antibodies during conditioning or (iii) in patients receiving prophylaxis with post-transplant cyclophosphamide (Pt-Cy) prophylaxis due to T-cell activation in the 3-day period between graft infusion and start of immunosuppression. Our group recently implemented a restrictive antibiotic protocol for patients likely to develop fever due to CRS following Anti-thymocyte Globulin (ATG) treatment, and reducing overall antibiotic use in the early ASCT period by 30% and its initiation by six days until true neutropenic fever emerged. Although simple laboratory markers to differentiate CRS-induced FUO from infectious fever remain elusive, emerging molecular techniques to detect microbiota may soon improve diagnostics. Conversely, strategies such as a short course of systemic antibiotics or other approaches investigated in murine models so far may specifically suppress bacterial translocation in new onset GvHD and disrupt the vicious inflammatory circle in GvHD, though definitive clinical indicators of translocation are missing in daily clinical practices and clinical trials are needed to validate this approach. Antibiotic stewardship may be supplemented by additional strategies to restore diversity such as repeated fecal microbiota transfer, dietary interventions or approaches neutralizing antibiotics selectively in the gastrointestinal tract by β-lactam cleaving enzymes and charcoal-based adsorbents or engineered probiotics. Finally, we also have to be aware, that non-antibiotic drugs frequently used in the setting of SCT additionally interfere with diversity and may need consideration [14]. Nevertheless, recognizing the antibiotic paradox in the clinical situation can sharpen physicians’ commitment to antibiotic stewardship, an essential strategy amid rising multidrug resistances.
